# Treatment With Inhaled Nitric Oxide and General Intelligence in Preterm Children in Two European Cohorts

**DOI:** 10.1111/apa.70118

**Published:** 2025-05-06

**Authors:** Nicole Tsalacopoulos, Valérie Benhammou, Laetitia Marchand‐Martin, Véronique Pierrat, Pierre‐Yves Ancel, Armita Shahesmaeilinejad, Viktoria Rücker, Vincent Prevot, Konstantina Chachlaki, Christoph Härtel, Wolfgang Göpel, Juliane Spiegler, the miniNO Consortium

**Affiliations:** ^1^ Department of Pediatrics University Hospital Würzburg Würzburg Germany; ^2^ School of Psychological Sciences Monash University Melbourne Australia; ^3^ Department of Population Health Sciences University of Leicester Leicester UK; ^4^ Department of Psychology University of Warwick Coventry UK; ^5^ Epidemiology and Statistics Research Center/CRESS INSERM, INRAE, Paris Cité University Paris France; ^6^ Department of Neonatal Medicine Créteil Hospital Créteil France; ^7^ Clinical Research Unit, Center for Clinical Investigation P1419 Assistance Publique Hôpitaux de Paris Paris France; ^8^ Institute for Medical Data Science University Hospital Würzburg Würzburg Germany; ^9^ Institute of Clinical Epidemiology and Biometry Julius‐Maximilians‐University of Würzburg Würzburg Germany; ^10^ Laboratory of Development and Plasticity of the Neuroendocrine Brain, Lille Neuroscience & Cognition University Lille, Inserm, CHU Lille Lille France; ^11^ Hospital University Federation (FHU) 1000 First Days of Life University Lille, Inserm, CHU Lille Lille France; ^12^ University Research Institute of Child Health and Precision Medicine National and Kapodistrian University of Athens, “Aghia Sophia” Children's Hospital Athens Greece; ^13^ Department of Pediatrics University Hospital Schleswig‐Holstein, Campus Lübeck Lübeck Germany

**Keywords:** cognitive function, inhaled nitric oxide, IQ, minipuberty, preterm birth

## Abstract

**Aim:**

To investigate whether treatment with inhaled nitric oxide is associated with cognitive performance at age 5–6 years in preterm‐born children.

**Methods:**

We analysed preterm children from two large European cohort studies, the German Neonatal Network (GNN) (*N* = 3606) and the French EPIPAGE‐2 cohort (*N* = 2579) admitted to neonatal care and followed up at age 5–6 years. Both cohorts had recorded data on iNO treatment. General cognitive ability was tested with IQ tests. Classification and Regression trees analysis was used to identify prenatal, perinatal and neonatal, clinical and social‐environmental predictors of IQ.

**Results:**

In both cohorts, treatment with inhaled nitric oxide was not associated with IQ at age 5–6 years. Analysis identified maternal educational level, gestational age at discharge from hospital, intraventricular haemorrhage and maternal country of birth as important factors associated with IQ scores.

**Conclusion:**

Treatment with inhaled nitric oxide was neither negatively nor positively associated with IQ at age 5–6 years. Neonatal and brain health, as well as socioeconomic factors are important for cognitive performance in early childhood.

**Summary**

- This study examined the association of inhaled nitric oxide (iNO) with cognitive function at 5–6 years in preterm children in two European birth cohorts.
- About 3%–4% of infants received iNO in the German and French cohort respectively.
- While treatment with iNO was not associated with cognitive function at 5–6 years, neonatal health and socioeconomic factors were important predictors.

**Abbreviations**
BPD: bronchopulmonary dysplasia
CART: classification and regression trees
EPIPAGE‐2: Etude épidémiologique sur les Petits Ages Gestationnels‐2
FSH: follicle‐stimulation hormone
GNN: German Neonatal Network
iNO: inhaled nitric oxide
IQ: intelligence quotient
IVH: intraventricular haemorrhage
LH: luteinising hormone
NICU: neonatal intensive care units
WPPSI: Wechsler Preschool and Primary Scale of Intelligence

## 1 Introduction

Compared with term‐born children, preterm infants (born < 37 weeks of gestation) are at particular risk for respiratory distress and pulmonary hypertension. A recent meta‐analysis showed that about 24% of preterm infants develop early pulmonary hypertension [1]. Among extremely preterm‐born children, the rate of persistent pulmonary hypertension has been reported within a large range from 3% to 55% [1, 2]. Being small for gestational age, deficiency of amniotic fluid, clinical chorioamnionitis and premature rupture of membranes and incomplete treatment with antenatal steroids have been associated with early pulmonary hypertension [1, 2].

Nitric oxide (NO) is an important regulator of vascular tone [3]. Treatment with inhaled NO (iNO) increases NO availability in the pulmonary system and is therefore used as a routine therapy for pulmonary hypertension, in particular in infants born at or near term [4]. The evidence for treatment with iNO in preterm populations, however, is less clear. While some studies have investigated iNO as early routine or rescue therapy, evidence suggests limited efficacy of iNO treatment in this population for preventing serious brain injury and adverse neurodevelopmental outcomes, and survival without bronchopulmonary disease [5]. iNO treatment for preterm born infants is therefore currently discouraged or not outright recommended [6].

Recent animal studies have shown that NO is implicated in the regulation of postnatal activation of the hypothalamic–pituitary‐gonadal axis, also known as minipuberty [7]. Minipuberty leads to increasing levels of luteinising hormone (LH) and follicle‐stimulation hormone (FSH) through gonadotropin‐releasing hormone neurons of the hypothalamus [8]. Importantly, this hormonal surge seems to be higher and longer in preterm‐born infants compared with term‐born individuals [9]. Based on previous work describing the biological mechanisms between iNO, minipuberty and cognitive function [7, 8, 9], the overarching objective of the ongoing European miniNO project (https://minino‐project.com) is to investigate whether prolonged treatment with iNO can regulate the surge of LH and FSH during minipuberty in preterm‐born infants, and thus positively affect brain development, including cognitive function [7, 10].

Given that preterm‐born infants are treated with iNO in clinical practice, the aim of the present study was to investigate whether iNO treatment is associated with the cognitive performance of preterm children aged 5–6 years by utilising already existing data. Data on treatment with iNO and cognitive performance, assessed through IQ tests, were drawn from two large cohort studies in Germany and France. Other social‐environmental, perinatal, treatment and neonatal factors that are implicated in the treatment with iNO, cognitive performance and preterm birth were available in both cohort studies.

## 2 Methods

### 2.1 Participants

#### 2.1.1 GNN

**FIGURE 1:**
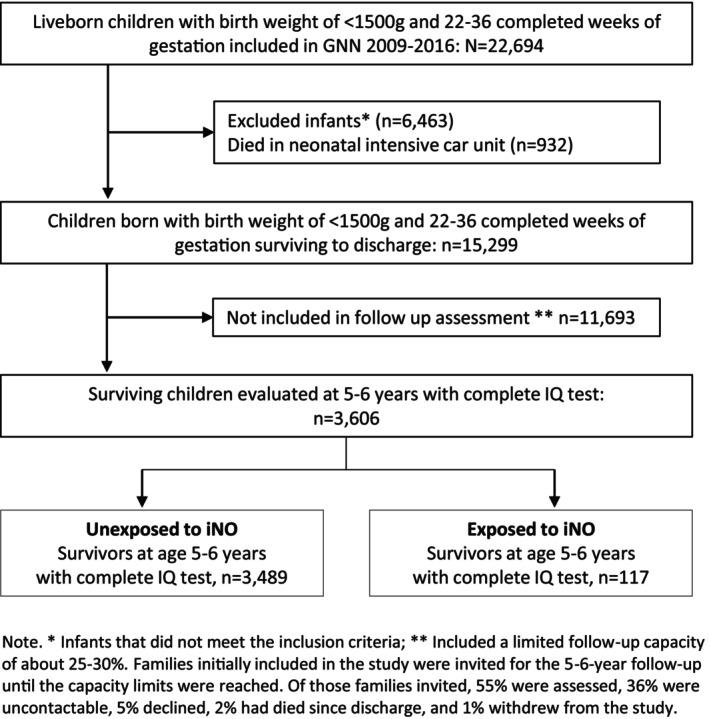
Flow diagram of the participant recruitment and selection process for the GNN 2009–2016 cohort study.

The German Neonatal Network (GNN) is a population‐based observational multicentre cohort study of preterm infants born between 1 January 2009 and 31 December 2016 who were transferred to one of 68 neonatal intensive care units (NICUs) in Germany. All infants were born with a birth weight < 1500 g and a gestational age of 22–36 weeks. Due to study design restrictions, about 30%–40% of infants initially enrolled in the study were invited for follow‐up at 5–6 years. Details of the recruitment have been described elsewhere [11]. However, of those invited for follow‐up, 55% were examined, with data on IQ available for 3606 children (see Figure 1).

Approval was given by the local ethics committee for research in human subjects at the University of Lübeck (# 08–022) and by the local ethics committees of all participating centres. Written informed consent was obtained from the infants' parents or legal guardians.

#### 2.1.2 EPIPAGE‐2

**FIGURE 2:**
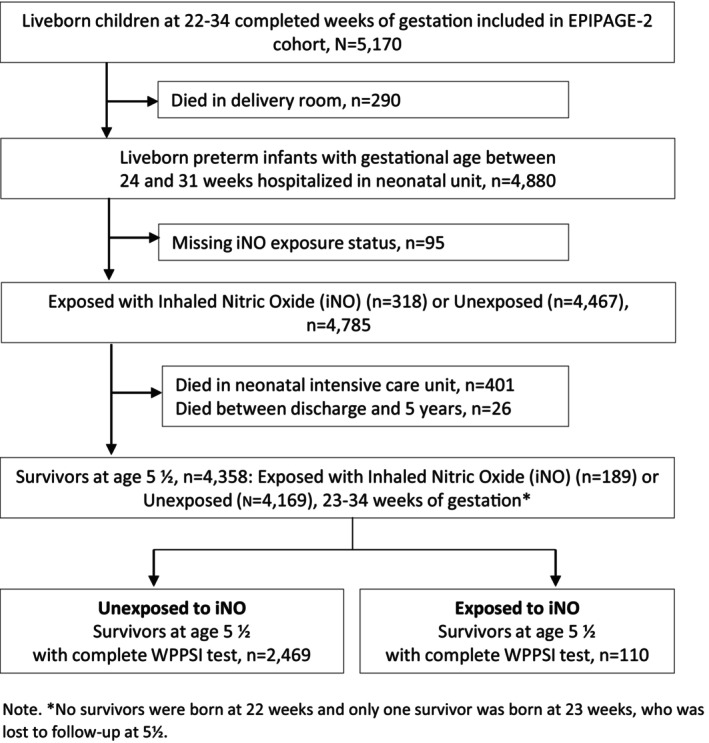
Flow diagram of the participant recruitment and selection process for the EPIPAGE‐2 cohort study.

The etude épidémiologique sur les petits ages gestationnels‐2 (EPIPAGE‐2) is a French prospective national population‐based cohort study. Children and their families were recruited in 2011 with details described elsewhere [12]. All births at 22–34 weeks of gestation were eligible for inclusion, and survivors were invited to participate in a neurodevelopmental assessment at 5 years. Data on IQ were available for 2579 children (Figure 2).

The EPIPAGE‐2 study was approved by the National Data Protection Authority (CNILDR‐2011‐089, DR‐2016‐290), the Consultative Committee on Treatment of Information on Personal Health Data for Research Purposes (no. 16.626 and 16.263) and the Committee for Protection of People Participating in Biomedical Research (no. 2011‐A00159‐32 and 2016‐A00333‐48). Children whose parents had agreed to participate at birth were eligible for follow‐up. Written informed consent from both parents was required for the 5.5‐year assessment.

### 2.2 Cognitive Outcome

General cognitive performance was assessed using the Wechsler Preschool and Primary Scale of Intelligence (WPPSI) (GNN: third version; EPIPAGE‐2: fourth version). The WPPSI was administered by trained study nurses in the GNN and by psychologists in the EPIPAGE‐2 study.

### 2.3 Potential Predictors and Risk Factors

#### 2.3.1 Variable of Interest

iNO treatment was defined as infants treated with iNO (=1) versus infants not treated with iNO (=0).

The following known social‐environmental, perinatal, treatment and neonatal factors were considered:

#### 2.3.2 Social‐Environmental

Maternal age at delivery (in years), maternal educational level (postsecondary school or higher), foreign country of birth (yes/no).

#### 2.3.3 Perinatal

Pre‐eclampsia (yes/no), premature rupture of membranes (yes/no), antenatal steroids (yes/no), infant sex (male/female), admission temperature (in degrees Celsius), gestational age (in weeks), birth weight (Fenton *z*‐score).

#### 2.3.4 Treatment

Mechanical ventilation (in days), parenteral nutrition (in days), postnatal steroids (yes/no), midazolam [13] (yes/no).

#### 2.3.5 Neonatal

Intraventricular haemorrhage (IVH; 0 = None, 1 = Grade 1 or 2; 2 = Grade 3 or 4), bronchopulmonary dysplasia (BPD; defined as supplemental oxygen or any respiratory support required at 36 weeks postmenstrual age; yes/no), episodes of clinical sepsis (0 = no episode, 1 = 1 or 2 episodes, 2 = 2 or more episodes) and retinopathy of prematurity (0 = None, 1 = Grade 1 or 2, 2 = Grade 3 or 4). Gestational age (in weeks) at discharge was considered as a proxy for level of morbidity; preterm infants with severe or more complications tend to be discharged at a more mature gestational age compared with those who have an uncomplicated clinical course [14].

### 2.4 Statistical Analysis

The analyses were carried out using SPSS and R software. Differences between infants treated and not treated with iNO were tested with a Chi‐square test for categorical variables or t‐test for continuous variables. To ensure representativeness, EPIPAGE‐2 percentages and means were weighted to account for the cohorts sampling design. Children born at 22–26 weeks' gestation were recruited over 8 months (i.e., equivalent of 35 weeks), those born at 27–31 weeks' gestation over 6 months (i.e., 26 weeks) and those born moderately preterm, at 32–34 weeks' gestation, over 5 weeks [15]. The corresponding weights were 1 for children born at 24–26 weeks' gestation, 1.34 (35/26) for those born at 27–31 weeks' gestation and 7 (35/5) for those born at 32–34 weeks' gestation.

To identify predictors of IQ we used a decision tree method, that is, classification and regression trees (CART) analysis [16] with R software, Version 4.2.1 and the rpart package [17]. All potential predictors and risk factors (i.e., iNO treatment, maternal age at delivery, educational level and country of birth, pre‐eclampsia, premature rupture of membranes, antenatal steroids, infant sex, admission temperature, gestational age, birth weight, mechanical ventilation, parenteral nutrition, postnatal steroids, midazolam, IVH, BPD, episodes of clinical sepsis, retinopathy of prematurity, gestational age at discharge) and IQ were included in the CART analysis, allowing the algorithm to automatically select and account for the complex interactions among all variables, including their potential confounding effects. The decision tree model was trained using the rpart function with an ANOVA method. To ensure an interpretable and generalisable model, a minimum of 30 observations in every node and a minimum of 15 observations in every terminal node (leaf) were set. The rpart.control() function was applied to set stopping rules and to ensure the robustness of the prediction model. To refine the model and to avoid overfitting the complexity parameter was optimised. This parameter controls the pruning of the tree by setting a threshold for the minimum improvement needed for a split to be retained. The initial model was fitted with a default value of 0.01. Subsequently, a 10‐fold cross‐validation was used to identify the parameter value that minimised the cross‐validated error. The optimal value was used to prune the tree, ensuring that only meaningful splits were retained.

To describe the strength of the associations between all potential predictors and risk factors (i.e., iNO treatment, maternal age at delivery, educational level and country of birth, pre‐eclampsia, premature rupture of membranes, antenatal steroids, infant sex, admission temperature, gestational age, birth weight, mechanical ventilation, parenteral nutrition, postnatal steroids, midazolam, IVH, BPD, episodes of clinical sepsis, retinopathy of prematurity, gestational age at discharge) and IQ at 5–6 years, a multivariable regression model was employed (see Table S1).

## 3 Results

Perinatal, neonatal and social‐environmental characteristics of both cohort studies are presented according to iNO treatment in Table 1.

**TABLE 1:** Characteristics of the GNN and EPIPAGE‐2 cohort studies stratified by iNO treatment.

Variables	Total		GNN 2009–2016 (*N* = 3606)					Total		EPIPAGE‐2 (*N* = 2579)				
			No iNO		iNO		*p*			No iNO		iNO		*p*
			*N* = 3489		*N* = 117					*N* = 2469		*N* = 110		
**Social‐environmental**														
Maternal age at delivery (in years), mean (SD)	31.7	(5.4)	31.7	(5.4)	31.9	(5.6)	0.35	30.7	(5.4)	30.7	(5.4)	30.6	(6.1)	0.49
Maternal education, postsecondary or higher, *n* (%)	1622	(48.3)	1577	(48.6)	45	(40.5)	0.11	1371	(56.3)	1320	(56.4)	51	(49.2)	0.21
Foreign country of birth, *n* (%)	612	(18.6)	584	(18.3)	28	(25.5)	0.07	2023	(15.0)	440	(15.0)	23	(19.6)	0.21
**Perinatal**														
Pre‐eclampsia, *n* (%)	265	(7.4)	262	(7.5)	3	(2.6)	0.04	441	(15.2)	420	(15.1)	21	(18.5)	0.36
Premature rupture of membranes, *n* (%)	1097	(31.0)	1032	(30.1)	65	(57.0)	< 0.001	930	(37.0)	877	(36.8)	53	(43.7)	0.20
Antenatal steroids, *n* (%)	3304	(91.7)	3200	(91.8)	104	(88.9)	0.34	2102	(79.4)	2014	(79.4)	88	(78.2)	0.81
Sex male, *n* (%)	1868	(51.8)	1802	(51.6)	66	(56.4)	0.31	1351	(53.9)	1298	(54.0)	53	(50.1)	0.49
Admission temperature (in degree Celsius), mean (SD)	36.7	(0.8)	36.7	(0.7)	36.5	(1.1)	0.03	36.5	(1.0)	36.5	(1.0)	36.1	(0.9)	< 0.001
Gestational age (in weeks), mean (SD)	27.7	(2.4)	27.7	(2.4)	25.8	(2.1)	< 0.001	31.7	(2.4)	31.8	(2.4)	28.0	(2.5)	< 0.001
Birth weight (Fenton z‐score), mean (SD)	−0.4	(0.9)	−0.4	(0.9)	−0.3	(1.0)	0.06	−0.3	(0.8)	−0.3	(0.8)	−0.2	(0.8)	0.25
**Treatment**														
Mechanical ventilation (in days), median (IQR)	1	(8)	1	(7)	13	(23)	< 0.001	2.2	(6.8)	1.8	(5.9)	19.0	(16.6)	< 0.001
Parenteral nutrition (in days), median (IQR)	14	(10)	13	(10)	18	(12)	< 0.001	11.5	(10.2)	11.2	(9.8)	26.8	(15.4)	< 0.001
Postnatal steroids, *n* (%)	558	(15.5)	497	(14.2)	61	(52.1)	< 0.001	165	(2.8)	126	(2.2)	39	(32.4)	< 0.001
Midazolam, *n* (%)	643	(17.9)	599	(17.2)	44	(37.6)	< 0.001	395	(9.5)	328	(8.4)	67	(63.7)	< 0.001
**Neonatal**														
Gestational age at discharge (in weeks), mean (SD)	38.8	(3.6)	38.7	(3.4)	41.8	(6.5)	< 0.001	37.4	(2.5)	37.3	(2.3)	41.1	(5.4)	< 0.001
IVH, *n* (%)							< 0.001							< 0.001
None	2971	(82.4)	2897	(83.0)	74	(63.8)		1819	(81.1)	1768	(81.8)	51	(51.2)	
Grade 1 or 2	423	(11.7)	402	(11.5)	21	(18.1)		662	(17.3)	617	(16.9)	45	(36.4)	
Grade 3 or 4	211	(5.9)	190	(5.4)	21	(18.1)		72	(1.6)	58	(1.4)	14	(12.4)	
BPD, *n* (%)	692	(19.2)	631	(18.1)	61	(52.1)	< 0.001	229	(4.1)	182	(3.3)	47	(41.9)	< 0.001
Of those:														
Moderate BPD	549	(79.3)	512	(81.1)	37	(60.7)		192	(84.8)	158	(87.8)	34	(73.5)	
Severe BPD^a^	143	(22.7)	119	(18.9)	24	(39.3)		37	(15.2)	24	(12.2)	13	(26.5)	
Retinopathy of prematurity, *n* (%)														
None	2124	(62.7)	2083	(63.5)	41	(36.6)	< 0.001	2381	(97.0)	2293	(97.3)	88	(84.9)	< 0.001
Grade 1 or 2	1047	(30.9)	997	(30.4)	50	(44.6)		156	(2.6)	145	(2.5)	11	(8.8)	
Grade 3 or 4	219	(6.5)	198	(6.0)	21	(18.8)		24	(0.4)	15	(0.2)	9	(6.4)	
Episodes of clinical sepsis, *n* (%)														
None	3137	(87.0)	3037	(87.0)	100	(85.5)	0.76	2106	(91.3)	2049	(91.9)	57	(58.3)	< 0.001
1	395	(11.0)	380	(10.9)	15	(12.8)		322	(6.7)	289	(6.2)	33	(29.2)	
≥ 2	74	(2.0)	72	(2.1)	2	(1.7)		124	(2.1)	109	(1.9)	15	(12.4)	
**Cognitive outcome at 5–6 years**														
IQ, mean (SD)	98.6	(14.2)	98.7	(14.1)	93.8	(16.5)	0.005	98.7	(14.5)	98.9	(14.4)	89.0	(18.0)	< 0.001

*Note:* Continuous variables are given as mean and standard deviation (SD) or median and interquartile range (IQR), and categorical variables as number and percentage; percentages and means for EPIPAGE‐2 are weighted to account for differences in survey design between gestational age groups.

Abbreviations: BPD, bronchopulmonary dysplasia; iNO, inhaled nitric oxide; IQ, intelligence quotient; IVH, intraventricular haemorrhage.

^a^ Received oxygen at discharge.

### 3.1 GNN

In the GNN cohort, 3.2% of children (*n* = 117) received iNO. Children who received iNO had similar social‐environmental and maternal characteristics compared with those not treated with iNO. Mothers of children who received iNO less often experienced pre‐eclampsia but more often premature rupture of membranes. While the admission temperature and gestational age of infants with iNO treatment were lower compared with infants without iNO treatment, their birth weight was slightly higher. Infants who received iNO more likely received other treatments, such as mechanical ventilation, parenteral nutrition, postnatal steroids and midazolam. Neonatally, children treated with iNO more often had IVH and BPD and were discharged at a higher gestational age than children not treated with iNO. At 5–6 years, children who received iNO had a lower IQ score than those without iNO treatment.

### 3.2 EPIPAGE‐2

Within the EPIPAGE‐2 cohort, 2.0% (weighted percent, *n* = 110) infants received iNO. There was no difference in maternal and social‐environmental characteristics between children who received iNO and those who did not. Children treated with iNO had a lower admission temperature and a lower gestational age at birth compared with infants not treated with iNO. In addition, children who received iNO were more likely to be treated with steroids and midazolam, and received mechanical ventilation and parenteral nutrition for a longer period of time. Further, infants treated with iNO were more likely to be discharged at a higher gestational age and to experience IVH, BPD, retinopathy of prematurity and episodes of clinical sepsis. At 5–6 years, children treated with iNO had a lower IQ than children not treated with iNO.

## 4 Is iNO Treatment Associated With IQ at Age 5–6 Years?

### 4.1 GNN

**FIGURE 3:**
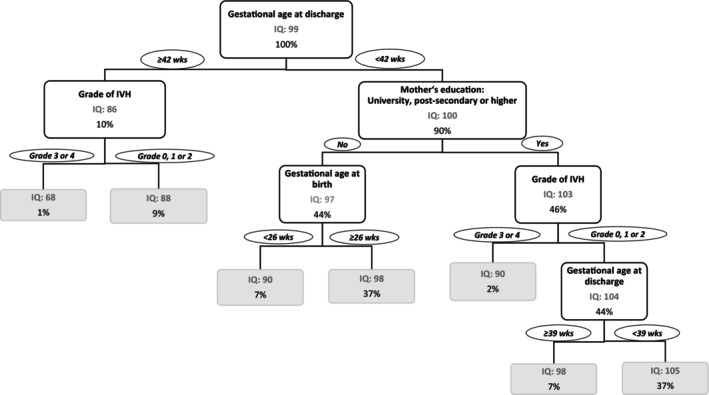
Classification tree resulting from RPART analysis of early predictors and risk factors associated with IQ at the age of 5–6 years based on GNN 2009–2016 data. *Note:* Important early factors associated with IQ at age 5–6 years were gestational age at discharge of less than 39 weeks, maternal postsecondary school education and higher, no IVH (intraventricular haemorrhage) or IVH Grade 1 or 2. That is, those children that were discharged from the NICU before 39 weeks of gestational age, whose mother achieved an educational level of postsecondary school and who had no or a mild IVH were more likely to have an above average IQ score of 105 at age 5–6 years.

CART analysis (see Figure 3) showed that children with a gestational age at discharge of ≥ 42 weeks were more likely to have a mean IQ of 86 at 5–6 years, compared with those discharged at an earlier gestational age (< 42 weeks), who achieved a mean IQ of 100. Among infants discharged at ≥ 42 weeks of gestation (10% of all children), those with no or Grade 1 or 2 IVH had a higher mean IQ compared with those who experienced a severe IVH (Grade 3 or 4) (mean IQ of 88 and 68 respectively). A large proportion of children was discharged with a gestational age below 42 weeks (90%). About 7% of children who were discharged at less than 42 weeks gestational age, who had a mother with a low educational level (i.e., below postsecondary level) and who were born with a gestational age below 26 weeks had a mean IQ of 90, while 37% of children who were born with a gestational age ≥ 26 weeks achieved a mean IQ of 98. In contrast, 2% of children whose mother had a postsecondary or higher educational level and who experienced a severe IVH achieved a mean IQ of 90. But 7% of children with no or a mild IVH and who were discharged with a gestational age of ≥ 39 weeks had a mean IQ of 90. A mean IQ score of 105 was achieved by 37% of all children who had no or a mild IVH and who were discharged before 39 weeks' gestation.

### 4.2 EPIPAGE‐2

**FIGURE 4:**
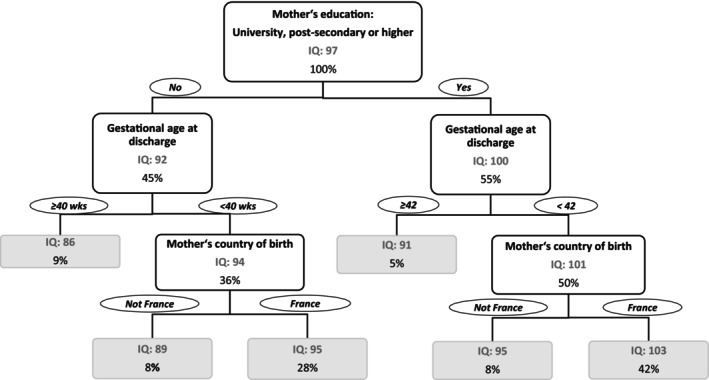
Classification tree resulting from RPART analysis of early predictors and risk factors associated with IQ at the age of 5–6 years based on EPIPAGE‐2 data. *Note:* Important early factors associated with IQ at age 5–6 years were maternal education of at least postsecondary school level, a gestational age at discharge of less than 42 weeks and France as the mother's country of birth. That is, children whose mother had a high educational level, who were discharged from the NICU before 42 weeks of gestational age and whose mother was born in France were more likely to have an above average IQ score of 103 at age 5–6 years.

Figure 4 describes the results from the CART analysis in the EPIPAGE‐2 cohort. About 45% of all children had a mother with a low educational level (i.e., lower than postsecondary school). These children had a mean IQ score of 92. Children who were also discharged at ≥ 40 weeks' gestation achieved a mean IQ of 86, while those with a gestational age at discharge of < 40 weeks and with a mother who was not born in France had a mean IQ of 89. In contrast, children whose mother was born in France achieved a higher mean IQ of 95 (28% of all children). Children who had a mother with a high educational level (University, postsecondary or higher) achieved a mean IQ of 100 at age 5–6 years. But children who were discharged at a later gestation age ≥ 42 weeks only had a mean IQ of 91. In contrast, children discharged earlier, at less than 42 weeks' gestation, and whose mother was not born in France had a mean IQ of 95. However, if the child's mother was born in France (about 42% of all children) the mean IQ was 103.

In both cohorts, the results from the CART analysis (with the multivariate effect from the linear regression models, Table S1) identified maternal educational level (GNN: estimate 5.5, 95% CI: 4.5, 6.5; EPIPAGE‐2: estimate 8.0, 95% CI: 6.8, 9.3), gestational age at discharge from hospital (GNN: estimate −0.5, 95% CI: −0.7, −0.3; EPIPAGE‐2: estimate −0.7, 95% CI: −1.0, −0.3), IVH (GNN: estimate 11.6, 95% CI: 9.1, 14.1; EPIPAGE‐2: estimate 2.6, 95% CI: −1.5, 6.7) and maternal country of birth (GNN: estimate 5.3, 95% CI: 4.0, 6.5; EPIPAGE‐2: estimate 6.7, 95% CI: 5.1, 8.3) as the most important predictors of IQ at 5–6 years over treatment with iNO (GNN: estimate 1.4, 95% CI: −1.5, 4.4; EPIPAGE‐2: estimate −3.0, 95% CI: −6.5, 0.6).

## 5 Discussion

This study of preterm‐born children in two European countries found no evidence that iNO treatment is associated with IQ at 5–6 years. Instead, maternal educational level, gestational age at discharge from hospital, IVH and maternal country of birth were identified as the most important factors associated with higher IQ scores.

The findings of this study are largely in accordance with previous studies that were predominately based on RCTs [5]. These studies investigated the effect of iNO on short‐term (up to 2 years) mortality and morbidity, including BPD, IVH [5, 18] but also on long‐term (age 5 and 7 years) neurodevelopmental and behavioural outcomes [19, 20]. None of these aforementioned studies were able to provide evidence for an association of iNO treatment with any of these short‐ and long‐term outcomes. However, a more recent observational study that included data from 11 countries [21], reported increased in‐hospital mortality for children treated with iNO compared with controls, but similarly to previous studies found no evidence for an association between iNO treatment and neurodevelopmental outcomes at age 5 years. Notably, the study by Siljehav and colleagues [21] used propensity score matching in order to balance baseline characteristics between participants who received iNO treatment and matched controls who did not receive iNO treatment. Although good covariate balance is provided by propensity score matching, CART analysis has the advantage of accounting for nonlinearity or highly skewed covariates [16], as well as for interactions and correlations between covariates [22].

In clinical practice, very ill preterm‐born infants are treated with iNO as an early routine or rescue therapy for respiratory problems, following persistent pulmonary hypertension [23]. Preterm‐born babies with pulmonary hypertension have a higher risk of further neonatal complications independent of iNO treatment [2], and lower cognitive scores are therefore expected. While iNO may balance or reduce the hormonal amplitude during minipuberty in preterm infants, the current study suggests that iNO treatment did not affect general cognitive performance, that is, intelligence at age 5–6 years—at least not over and above other peri‐ and neonatal factors, such as maternal education, gestational age at discharge and IVH.

Notably and largely consistent with previous work [24], our study shows that maternal educational level, gestational age at discharge from hospital/NICU, the presence of IVH and maternal country of birth are important factors for the cognitive function of preterm born children. Maternal educational level reflects socioeconomic status and has been found to be an important factor associated with IQ in general and preterm populations [24, 25]. In addition, in the EPIPAGE‐2 cohort, maternal birth in a foreign country was associated with IQ at age 5–6 years. The mothers of about 15% of preterm‐born infants were not born in France. In the GNN, a similar rate of mothers (17%) were not born in Germany. However, the mother's country of birth was not associated with IQ in the GNN cohort study, and health and antenatal care systems seem to be similar in both countries [26]. A previous study found that women born outside continental France had a higher risk of experiencing a perinatal death despite adjustments for age, parity and socioeconomic factors [27]. The authors speculate that the excess risk attributed to birthplace outside of France may be a residual variation in socioeconomic status [28], or may be explained by medical problems during pregnancy and delivery, and cultural practices [29]. Indeed, in the EPIPAGE‐2 cohort, most women born outside of France were from an African country (about 10%) while about 2% of mothers were born in other European countries [28]. In contrast, most GNN mothers born outside of Germany were born in another European country (approx. 9%), while 5% came from a Middle Eastern country, 1% from Asia and 3% from an African country.

Similar to SES, neonatal brain injury, including IVH, has been linked to poor cognitive and neurodevelopmental outcomes [25]. Interestingly, gestational age at discharge was an important predictor of IQ in both cohorts. While this association has not been reported previously, gestational age at discharge reflects the lengths of stay in hospital. Length of hospitalisation has been associated with poorer outcomes for preterm children, including cognitive performance [30]. In addition, lengths of stay in hospital can be interpreted as a measure of brain and overall health [30], severity of the clinical and neonatal course, speed of recovery and resilience of the infant. In contrast to gestational age at discharge, gestational age at birth was less important for a higher IQ score in preterm children. In the GNN, being discharged before 42 weeks of gestation, having a mother with a lower level of education, and being born before 26 weeks of gestation was associated with a lower mean IQ score. In the EPIPAGE‐2, gestational age at birth was not identified as a factor associated with IQ. Thus, it may be that the degree of prematurity is accounted for by other health and morbidity measures, such as IVH and gestational age at discharge.

Does that mean that iNO does not influence minipuberty? This is a question the current study cannot answer directly as data on hormone levels during the time of minipuberty were not available. However, this does not negate a balancing of hormone levels through the treatment with iNO, which as a result affects cognitive performance. iNO is typically administered only during the initial 48 h after birth, which may be insufficient to significantly affect minipuberty, a process that develops over several weeks and peaks at around 1–3 months of age [8]. Additionally, infants receiving iNO, whether as routine care or rescue therapy, may have been too critically ill for the treatment to effectively modulate the hormonal surges associated with minipuberty. Furthermore, any potential effects of iNO on cognitive outcomes seem to be overshadowed by other more significant predictors identified in the study.

The strengths of this study are its prospective longitudinal design, large sample size, and the use of a range of prenatal, perinatal and neonatal, clinical and social‐environmental factors, as well as the use of the same widely employed and reliable measure of general cognitive ability in both cohort studies. Given that the current study evaluates associations between iNO and other perinatal factors and IQ separately in both cohort studies, bias due to the use of different versions of the WPPSI is negligible. Importantly, the current study provides a uniquely built‐in replication with the same data in two longitudinal cohort studies from different countries. There are also limitations. Of all infants and families in the GNN study invited for follow‐up, only 55% were examined and administered an IQ test at 5–6 years. In EPIPAGE‐2, the follow‐up rate, including an IQ assessment at age 5–6 years, was higher, with about 60%. Further, iNO treatment is predominantly given to very sick infants who are more likely to die before discharge in both cohorts. Moreover, it is possible that other unmeasured factors could have influenced the here investigated associations, for example, genetic information, paternal education, parenting behaviour and home environment [31, 32, 33] or implementation of care and intervention [34]. Another limitation is the lack of detailed information on clinical indication, timing, dosage and duration of iNO treatment in both cohort studies included here. For instance, Chachlaki and colleagues [7] proposed that prolonged iNO treatment could have a beneficial effect on preterm children's brain development and cognitive performance. In this vein, Feng and colleagues [35] suggest that the effect of iNO depends on the age of the infant, birth weight and duration and dose of iNO treatment.

Overall, the factors that play a role in the association between preterm birth, minipuberty, treatment with iNO and later cognitive outcomes may be too complex [36], particularly in the group of very sick preterm children, making it difficult to quantify the effect of iNO treatment on cognitive performance. It is therefore important to investigate and test the associations between iNO treatment, minipuberty and long‐term cognitive outcomes in a study that offers information and data on all these factors and outcomes. The European miniNO project (https://minino‐project.com) is currently carrying out investigations across different studies and cohorts [7] in order to test the hypothesis that iNO treatment can regulate minipuberty in preterm born infants and thus positively affect sensory and cognitive function [7, 10].

Our results show that treatment with iNO is neither negatively nor positively associated with IQ at 5–6 years. However, the findings confirm previous work and emphasise the importance of neonatal and brain health, as well as socioeconomic factors for IQ in early childhood.

## Author Contributions

**Nicole Tsalacopoulos:** writing – review and editing, writing – original draft, formal analysis, investigation, visualization. **Valérie Benhammou:** formal analysis, writing – review and editing, investigation. **Laetitia Marchand‐Martin:** formal analysis, writing – review and editing, investigation. **Véronique Pierrat:** writing – review and editing, investigation. **Pierre‐Yves Ancel:** writing – review and editing, funding acquisition, investigation. **Armita Shahesmaeilinejad:** writing – review and editing, visualization, formal analysis, investigation. **Viktoria Rücker:** visualization, writing – review and editing, formal analysis, investigation. **Vincent Prevot:** writing – review and editing, investigation, funding acquisition. **Konstantina Chachlaki:** writing – review and editing, investigation, funding acquisition. **Christoph Härtel:** writing – review and editing, investigation. **Wolfgang Göpel:** writing – review and editing, funding acquisition, investigation. **Juliane Spiegler:** funding acquisition, writing – review and editing, conceptualization, investigation, writing – original draft, formal analysis, project administration, methodology, visualization, supervision.

## Conflicts of Interest

The authors declare no conflicts of interest.

## Supporting information


Appendix S1.
