# Supplementary material for: Treatment With Inhaled Nitric Oxide and General Intelligence in Preterm Children in Two European Cohorts
Source: Acta Paediatr. 2025 May 6;114(9):2346–56. doi: 10.1111/apa.70118 (PMC12336939; doi:10.1111/apa.70118)
Supplement: Supplementary file 1 — Appendix S1. [file APA-114-2346-s001.docx]

**Supplementary Material**

Supplementary Table S1. Univariate and multivariate associations between all potential predictors and risk factors and IQ at 5-6 years in the GNN and EPIPAGE-2 cohort studies.

|  | **GNN 2009-2016** | |  | **EPIPAGE-2** | |
| --- | --- | --- | --- | --- | --- |
|  | IQ at 5-6 years | |  | IQ at 5-6 years | |
|  | Univariate effect  (95% CI) | Multivariate effect  (95% CI) |  | Univariate effect  (95% CI) | Multivariate effect  (95% CI) |
| **iNO treatment** | -4.9 (-7.8, -2.0) | 1.4 (-1.5, 4.4) |  | -7.8 (-11.0, -5.0) | -3.0 (-6.5, 0.6) |
| **Social-environmental** |  |  |  |  |  |
| Maternal age at delivery (in years) | 0.3 (0.2, 0.4) | 0.2 (0.1, 0.3) |  | 0.2 (0.1, 0.3) | 0.1 (-0.0, 0.2) |
| Maternal education, post-secondary or higher | 7.0 (6.0, 8.0) | 5.5 (4.5, 6.5) |  | 8.6 (7.5, 9.7) | 8.0 (6.8, 9.3) |
| Country of birth (GNN: Germany, EPIPAGE-2: France) | 6.6 (5.3, 7.9) | 5.3 (4.0, 6.5) |  | 7.6 (6.1, 9.1) | 6.7 (5.1, 8.3) |
| **Perinatal** |  |  |  |  |  |
| Pre-eclampsia | 2.0 (0.1, 3.8) | 1.6 (-0.2, 3.5) |  | -0.5 (-2.0, 1.1) | 0.6 (-1.2, 2.4) |
| Premature rupture of membranes | -0.1 (-1.1, 1.0) | 0.2 (-0.9, 1.3) |  | 0.8 (-0.4, 2.0) | 0.2 (-1.1, 1.6) |
| Antenatal steroids | 2.8 (1.0, 4.7) | 1.3 (-0.6, 3.2) |  | 1.8 (0.2, 3.3) | 1.8 (0.1, 3.5) |
| Sex, male | -1.4 (-2.4, -0.4) | -1.8 (-2.8, -0.8) |  | 0.3 (-0.9, 1.4) | -0.7 (-2.0, 0.6) |
| Admission temperature (in degree Celsius) | 1.9 (1.2, 2.6) | 0.1 (-0.6, 0.8) |  | 0.5 (-0.0, 1.1) | 0.0 (-0.6, 0.6) |
| Gestational age (in weeks) | 1.5 (1.3, 1.7) | 0.8 (0.5, 1.1) |  | 1.0 (0.7, 1.2) | 0.6 (0.2, 1.0) |
| Birth weight (Fenton z-score) | 0.7 (0.2, 1.3) | 1.2 (0.5, 1.8) |  | 1.6 (0.9, 2.3) | 1.3 (0.4, 2.2) |
| **Treatment** |  |  |  |  |  |
| Mechanical ventilation (in days) | -0.3 (-0.4, -0.3) | -0.07 (-0.1, -0.0) |  | -0.3 (-0.3, -0.2) | -0.1 (-0.2, 0.1) |
| Parenteral nutrition (in days) | -0.2 (-0.2, -0.2) | -0.01 (-0.1, 0.0) |  | -0.2 (-0.2, -0.1) | 0.0 (-0.0, 0.1) |
| Postnatal steroids | -8.4 (-9.8, -7.0) | -1.2 (-2.8, 0.5) |  | -6.4 (-8.8, -4.1) | 0.3 (-2.9, 3.5) |
| Midazolam | -7.0 (-8.3, -5.7) | -2.3 (-3.7, -0.9) |  | -4.3 (-5.9, -2.7) | 0.3 (-1.8, 2.3) |
| **Neonatal** |  |  |  |  |  |
| Gestational age at discharge (in weeks) | -1.3 (-1.4, -1.1) | -0.5 (-0.7, -0.3) |  | -1.0 (-1.2, -0.8) | -0.7 (-1.0, -0.3) |
| IVH, Grade 0, 1 or 2 | 15.3 (13.0, 17.6) | 11.6 (9.1, 14.1) |  | 5.0 (1.4, 8.5) | 2.6 (-1.5, 6.7) |
| BPD | -8.7 (-10.0, -7.5) | -0.7 (-2.3, 1.0) |  | -6.1 (-8.2, -4.1) | 0.2 (-2.4, 2.9) |
| Retinopathy of prematurity | -5.4 (-6.4, -4.3) | -0.7 (-1.8, 0.5) |  | -5.4 (-7.7, -3.1) | -2.0 (-4.7, 0.7) |
| Episodes of clinical sepsis | -4.2 (-5.7, -2.7) | 0.5 (-1.1, 2.0) |  | -4.1 (-5.6, -2.6) | 0.6 (-1.4, 2.5) |

**Supplementary Table S1.** Univariate and multivariate associations between all potential predictors and risk factors and IQ at 5-6 years in the GNN and EPIPAGE-2 cohort studies.

Abbreviations: iNO=inhaled nitric oxide; IVH=intraventricular haemorrhage; BPD=bronchopulmonary dysplasia; IQ=intelligence quotient.
